# Exosome in Cardiovascular Diseases: A Complex World Full of Hope

**DOI:** 10.3390/cells8020166

**Published:** 2019-02-17

**Authors:** Gloria Bellin, Chiara Gardin, Letizia Ferroni, Juan Carlos Chachques, Massimo Rogante, Dinko Mitrečić, Roberto Ferrari, Barbara Zavan

**Affiliations:** 1 Department of Biomedical Sciences, University of Padova, 35131 Padua, Italy; gloria.bellin@gmail.com (G.B.); chiara.gardin@unipd.it (C.G.); letizia.ferroni@unipd.it (L.F.); 2 Maria Pia Hospital, GVM Care & Research, 10132 Torino, Italy; 3 Laboratory of Biosurgical Research (Alain Carpentier Foundation), Pompidu Hospital, University Paris Descartes, 75015 Paris, France; j.chachques-ext@aphp.fr; 4 Rogante Engineering Office, 62012 Civitanova Marche, Italy; main@roganteengineering.it; 5 Laboratory for Stem Cells, Croatian Institute for Brain Research, University of Zagreb School of Medicine, 10000 Zagreb, Croatia; dinko.mitrecic@mef.hr; 6 Maria Cecilia Hospital, GVM Care & Research, 48033 Ravenna, Italy; fri@unife.it

**Keywords:** exosomes, extracellular vesicle, cardiovascular disease

## Abstract

Exosomes are a subgroup of extracellular vesicles containing a huge number of bioactive molecules. They represent an important means of cell communication, mostly between different cell populations, with the purpose of maintaining tissue homeostasis and coordinating the adaptive response to stress. This type of intercellular communication is important in the cardiovascular field, mainly due to the fact that the heart is a complex multicellular system. Given the growing interest in the role of exosomes in cardiovascular diseases and the numerous studies published in the last few decades, we focused on the most relevant results about exosomes in the cardiovascular filed starting from their characterization, passing through the study of their function, and ending with perspectives for their use in cardiovascular therapies.

## 1. Introduction

Exosomes (EXOs) are a subgroup of extracellular vesicles (EVs), ranging in size from 30 to 100 nm, released by different cell types [1]. To date, they were found in numerous body fluids such as plasma, serum, saliva, amniotic fluid, breast milk, and urine; moreover, they are also released in cell culture media [2]. EXOs are rich in bioactive molecules, including DNA, messenger RNAs (mRNAs), microRNAs (miRNAs), and proteins; moreover, by transferring their bioactive cargos, they are important drivers in intercellular communication. 

**Figure 1:**
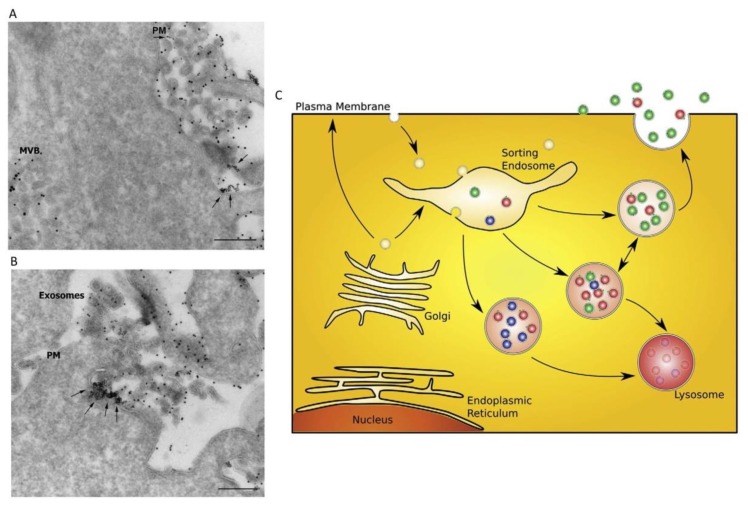
Release of exosomes (EXOs) and a sorting model. (**A**,**B**) Release of EXOs upon exocytic fusion of multivesicular bodies (MVBs) with the plasma membrane; arrows indicate the fusion profile. PM: plasma membrane; MVB: multivesicular body. Scale bars: 200 nm. (**C**) A proposed model for sorting of cargo into different MVB subpopulations. Different hypothetical MVB subclasses with distinct populations of intraluminal vesicles (red, green, and blue) are shown. Whether the MVBs contain a mixture of different intraluminal vesicles as depicted in the figure is not known. Sorting of cargo may already start within the biosynthetic pathway, at the plasma membrane, or within an endosomal compartment. Modified from Reference [1].

Many studies focused on the complex mechanisms of EXO biogenesis, secretion, and cargo loading; in fact, several steps are needed from the formation of intraluminal vesicles until fusion with the plasma membrane, passing through the loading of cargos. EXOs generate from the invagination of endosomes which form multivesicular bodies (MVBs) and they are secreted through the fusion of MVBs with the cell membrane; this process is mediated by the Rab family of proteins [3]. EXO composition is dependent on the cell type of origin, and is largely reflective of its physiological or pathological state. This means that the secretion, content, and function of EXOs strongly vary in response to changes in the cell microenvironment [4]. The precise selection of EXO cargo is not yet well clarified, although it seems that the protein sorting into EXOs is principally managed by endosomal sorting complexes required for transport (ESCRT) or by sphingolipids (ESCRT-dependent or ESCRT-independent pathways) [5,6]. Observations about RNA species suggested that some RNAs present motifs that may represent elements that favor their loading into EXOs [7] (Figure 1). Protected from the EXO lipid bilayer, protein and RNA cargo can persist in the extracellular space without undergoing degradation. Once secreted, EXOs can enter neighboring target cells or travel into the body fluids to even reach cells in distant districts; it was demonstrated that target cells internalize EXOs through a variety of methods such as endocytosis, membrane fusion, or ligand receptor binding, depending on source cells, target cells, and environments [8,9].

This evidence leads EXOs to become an attractive focus in biomedical research for investigating their role in different physiological and pathological settings as signaling mediators, biomarkers, and potential therapeutic targets. 

Recently, EXOs earned interest in the cardiovascular field due to the fact that, in a multicellular system, such as the heart, communication between different and not always close cells plays a fundamental role in the maintenance of physiological cardiac homeostasis and in the adaptive response to stress; EXOs are suitable candidates to have a central role in intercellular exchanges of information. It was demonstrated that they are involved in a wide range of cardiovascular processes, both physiological and pathological, with beneficial or detrimental activity [10,11,12,13].

**Figure 2:**
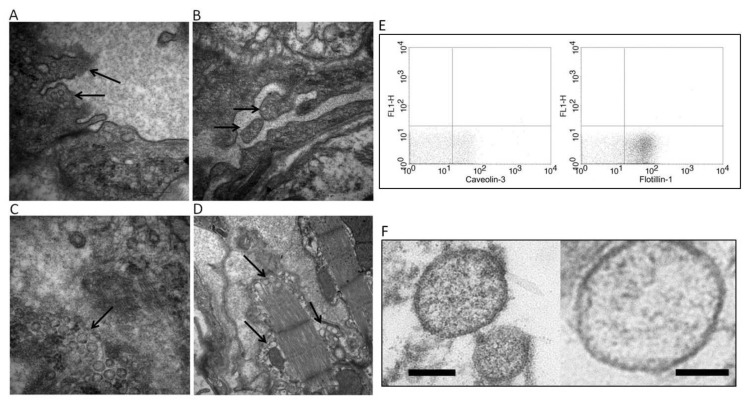
Electron microscopy of heart tissue. (**A**,**B**) Budding of the vessel wall to release the exosomes into the lumen (black arrows); (**C**) exosomes in the extracellular space; (**D**) exosomes released by myocyte fiber. Modified from Reference [16]. (**E**) Detection of proteins on cardiomyocyte (CM) EXO surface with flow cytometry. Mouse anti-caveolin-3 was detected on approximately 30% of EXOs (left), while mouse anti-flotillin-1 was detected on approximately 80% of EXOs. The distribution of EXOs presenting caveolin-3 and flotillin-1 indicates that the sample contains more than one population of EXOs. (**F**) Transmission electron microscopy of purified CM microvesicles (MVs)/EXOs. On the left, MVs/EXOs that display an electron-dense appearance are shown; on the right, MVs/EXOs that display an electron-lucent appearance are shown. Scale bar: 100 nm. Modified from Reference [15].

EXOs are released practically from all cells in the cardiovascular system and it was shown that stress conditions such as hypoxia or inflammation modulated their cargos and their release in conjunction with the target cells, contributing to improving or to impairing heart function [14] (Figure 2). Furthermore, Waldenström and colleagues highlighted the heterogeneity of microvescicles (MVs) and EXOs from the same cell type; in cardiomyocytes (CMs), 80% of the vesicles from the main cell population resulted positive for flotillin-1 surface antigen, while 30% were positive for carveolin-3, a protein exclusively present in cardiac muscle cells [15]. A part of them displayed an electron-dense appearance and another part displayed an electron-lucent interior. They hypothesized that these differences could imply a difference also in their cargo and target cells [15] (Figure 2). 

The multitude of cardiovascular processes in which EXOs are involved affords them great potential in the diagnostic and therapeutic fields as a novel alternative to whole-cell therapies. This is because they are more stable than cells, as well as being biocompatible and non-immunogenic; moreover, they are resistant to cryo-conservation without degrading [17]. However, it is necessary to say that EXO collection, isolation, and purification processes are still undergoing standardization. In fact, as highlighted by Tang and colleagues, different isolation methods lead to a discrepancy in purity, size, and concentration of EXOs and their cargo [18]. It follows that the populations of vesicles isolated with different methods are not homogeneous, and probably contain more than just exosomes. The lack of standardized methods can explain the difficulties in reproducibility and the discrepancies of results of some studies. 

In this review, we summarize the findings about the role of EXOs in the cross-talk between the different cardiac cell populations and their potential as diagnostic biomarkers and therapeutic means in cardiovascular diseases.

## 2. Exosomes from Different Cardiac Cells, Their Content, and Their Ability to Modulate Cell Behavior

A large part of heart volume is built by cardiac muscle, while the most abundant non-myocyte cell type is represented by cardiac fibroblasts (CFs) that account for about 90% of non-muscle cells. In contact with these cells, there are endothelial cells (ECs) that have important roles in cardiac homeostasis. Moreover, several studies highlighted the presence of resident cardiac-derived progenitor cells (CPCs) that are involved in the post-injury response [19,20]. The presence of such cells in the heart suggests the importance of heterocellular communication and the necessity to investigate the way in which this communication takes place. In this sense, EXOs are the main candidate for mediation between the various cell populations.

### 2.1. Cardiomyocytes

The first evidence that CMs release EXOs was provided by Gupta et al., who showed that EXOs were released both in physiological conditions and after hypoxia, with an increase in their release and in their content of heat-shock protein 60 (HSP60) under stress conditions [14]. A further study demonstrated that HSP60s contained in EXOs were bound to the membrane and were not released, preventing the pro-apoptotic effects induced by circulating HSP60 [10]. In the same study, proteomic analysis revealed that EXOs from primary CMs contained a pool of proteins that were common, but differed in amount, in EXOs obtained from CMs which underwent ethanol or hypoxia/reoxygenation stimuli. This pool included HSP60, tropomyosin-α, glyceraldehyde 3-phosphate dehydrogenase (GAPDH), myomesin, myosin-binding protein C, α-Cristallin B chain, and valosin-containing protein (VCP), while HSP27 and HSP90 were found only in hypoxia/reoxygenation-derived EXOs [10]. Moreover, EXOs from CMs that underwent hypoxia treatment seemed to promote apoptosis in nearby CMs due to the presence of tumor necrosis factor-α (TNF-α) in their cargo, favored by the activation of hypoxia-inducible factor-1α (HIF-1α) [12]. Another study demonstrated that hypoxic condition enriched EXOs in miRNA-30a that was efficiently transferred between CMs, favoring the maintenance of autophagic response probably through targeting *beclin-1* and the *Atg12* gene. Furthermore, CM transfection with a miRNA-30a inhibitor or a block of EXO release from CMs attenuated hypoxia-induced apoptosis [21]. Other miRNAs present in CM EXOs after hypoxia were found to be involved in the modulation of the apoptotic pathway; Zhang and colleagues indicated that rno-miR-21-5p, rno-miR-378-3p, rno-miR-152-3p, and let-7i-5p were upregulated after 48 h of hypoxia and, in particular, rno-miR-21-5p and rno-miR-378-3p appeared to have anti-apoptotic effects [11].

Since cardiovascular impairment is a major complication of diabetes, several studies focused on the involvement of EXOs in heart failure in diabetic conditions. For diabetic patients, physical exercise is important to decrease the possibility of developing cardiac dysfunction. Chaturvedi and colleagues studied EXOs released from cardiac muscle during exercise. They discovered that so-stimulated CM EXOs contained an elevated amount of mmu-mir-29b and mmu-mir-455, and that these miRNAs prevented the activation of matrix metalloproteinase 9 (MMP9), preserving the heart from the development of fibrosis and myocyte uncoupling [16]. This evidence served as a starting point to explore CM EXOs as a therapy for cardiac remodeling, since MMP9 inhibitors were not successful [16].

It was proven that EXOs from CMs could be internalized from other cells such as CFs and ECs, promoting the modulation of receiving cell behaviors. For example, the presence of CM EXO DNA in the CF cytosol and nucleus was shown, and this promoted gene expression modification. In particular, 175 genes were upregulated and 158 were downregulated in fibroblasts treated with CM EXOs [15]. A recent study indicated that the interaction between CMs and CFs is important in the progression of chronic heart failure, promoting the development of cardiac hypertrophy and dysfunction [22]. High expression of hsa-miR-217 in pathological rat CMs seemed to favor its release through EXOs that are taken up by CFs, promoting their proliferation and activation, and leading to heart fibrosis [22].

**Figure 3:**
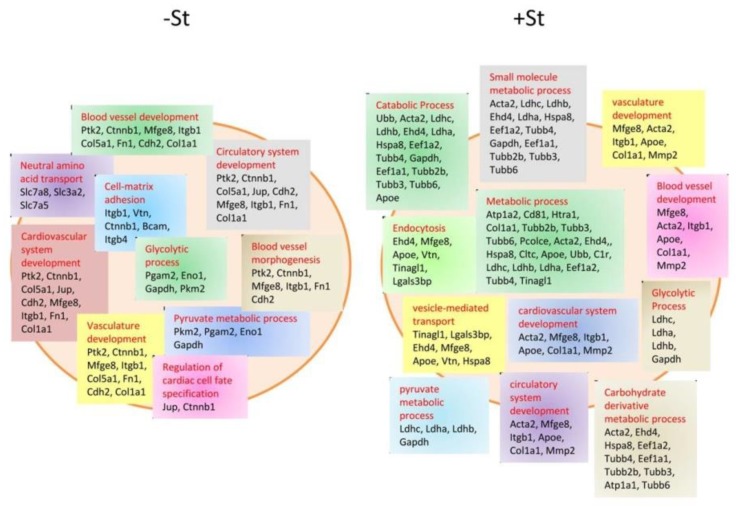
Schematic representation of protein content in EXOs from starved (+St), i.e., glucose-deprived, and non-starved (−St) CMs. CMs deprived of glucose change the protein pool contained in their EXOs, promoting their loading with proteins related to metabolic and catabolic processes, as well as blood vessel and cardiovascular development [24].

The close anatomical and functional relationship between CMs and ECs implicates the ability of CMs to communicate also with ECs and vice versa, above all during stress and pathological conditions. Wang et al. investigated the role of EXOs in CM and EC cross-talk in diabetic rats, showing that EXOs from pathological CMs were rich in rno-miR-320 and poor in rno-miR-126. This cargo modulated *insulin-like growth factor-1 (IGF-1)*, *HSP20*, and *Ets2* expression in ECs, promoting the downregulation of these genes; this seemed to lead to an inhibition of EC proliferation, migration, and tube-like formation [23]. On the contrary, deprivation of glucose, another stress condition, enhanced the release of EXOs from CMs with a glucose-dependent regulation of the cargo; CMs in normal culture conditions were shown to release EXOs that contained proteins mainly related to cell structure, growth, and survival, as well as mmu-miR-17, 20a, 23b, 30b, and 132. Contrariwise, CMs deprived of glucose produced EXOs rich in proteins involved in cell metabolism and in the proenergetic pathway, as well as mmu-miR-16, 17, 19a, 19b, 21, 23a, 23b, 30c, 125b-5p, 126-3p, 301a, and 301b [24] (Figure 3).

In particular, mmu-miR-17, 19a, 19b, 20a, 30c, and 126 were correlated with an increase in angiogenesis when internalized by ECs. This was demonstrated by Garcia et al., who showed a great propensity of EC cells to enter the synthesis (S) phase, and to increase tube formation when treated with starved-CM EXOs [24].

### 2.2. Cardiac Fibroblasts

**Figure 4:**
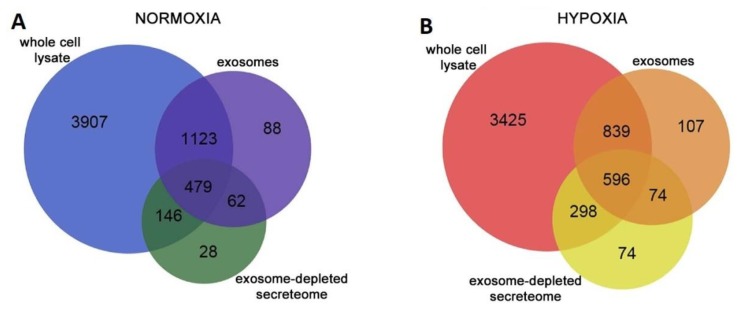
Summary of proteomics data acquired using a multidimensional protein identification technology approach. Venn diagrams representing the overlap of protein identifications between cardiac fibroblast (CF) whole-cell lysate, exosome, and secretome collected in (**A**) normoxic conditions and (**B**) hypoxic conditions. The number of proteins contained in the three fractions changed depending on the culture conditions. Modified from Reference [25].

CFs are the main cells involved in extracellular matrix (ECM) turnover, and, due to their secretory activity, they influence the physiology of other cells in the heart [25]. Despite this, only few works investigated CF EXO composition and activities. One was performed by Cosme and colleagues, who mapped and compared the proteomic profile of whole-CF lysate, CF secretome, and CF EXO content in normoxic and hypoxic conditions (Figure 4).

Focusing on EXOs, they found that normoxic and hypoxic conditions modified the number and the content of CF EXO proteins; under normoxic conditions, they identified 1752 proteins, while, following hypoxia, there were 1616 proteins. Moreover, comparing normoxia vs. hypoxia, 144 proteins resulted differentially expressed. Hypoxic conditions promoted EXO enrichment in ECM proteins such as multiple collagen type, perlecan, and fibronectin. Furthermore, they found an overrepresentation of mitochondria-associated proteins, and hypothesized that EXOs could be used by cells to remove dysfunctional mitochondria during stress conditions [25].

They also demonstrated that, depending on the time of co-treatment, CF EXOs had different effects on CM viability; if CF EXOs were added to CMs immediately before CM hypoxia treatment, they improved CMs viability; contrariwise, they reduced CM viability if added before the CM reoxygenation phase.

In addition to proteins, CF EXOs also contain miRNAs. It was shown that the 25.5% of fibroblast EXO miRNAs were represented by star miRNAs and, interestingly, Bang et al. discovered that fibroblasts were rich in rno-miR-21, while fibroblast-derived EXOs were enriched with rno-miR-21*. From their study, it seemed that rno-miR-21* was transported from CFs to CMs via EXOs, and that it could be involved in CM hypertrophy [26]. Tian and colleagues demonstrated that treatment with TNF-α favored the enrichment of CF EXOs with rno-miRNA-27a, rno-miRNA-28a, and rno-miRNA-34a. These miRNAs were transferred to CMs promoting the expression of hypertrophic markers such as atrial natriuretic peptide and β-myosin heavy chain in CMs [13].

The use of EXOs by CFs to communicate with CMs was also investigated by Lyu and colleagues, who noted that angiotensin II (AngII)-treated CFs stimulated the release of EXOs that were taken up by CMs. These EXOs in turn upregulated AngII expression together with the expression of its receptors in CMs, enhancing AngII-related cardiac hypertrophy [27].

From these studies, we can deduce that CFs utilize EXOs to communicate predominantly with CMs.

### 2.3. Endothelial Cells

Heart microvasculature is fundamental for cardiac health and cardiac tissue homeostasis. ECs, which form the endothelial barrier between blood and surrounding tissues, have a primary role in the maintenance of this homeostasis, especially following stress signals such as inflammation or hypoxia. The response of ECs to stress or damage signals leads not only to a release of growth factors and cytokines, but also EXOs that mediate their communication with each other and with the other cardiac compartments. 

A study carried out in 2012 showed that ECs cultured under different conditions released EXOs whose contents reflected cellular stress, varying in relation to the received stimuli [28]. Quantitative proteomics and mRNA arrays revealed that EXOs from ECs that underwent hypoxia or inflammation clearly differed from control EC EXOs; if stimulated with hypoxia, they presented a higher content of proteins involved in ECM remodeling, such as fibronectin and collagen, and an enrichment in mRNAs linked to stress response and apoptosis genes; if treated with TNF-α to mimic inflammation, EXOs resulted enriched in several factors concerning superoxide protection, immune response, and nuclear factor κB (NF-κB) pathway [28].

EXOs are used by ECs to communicate with each other and, particularly, to manage angiogenesis. It was found that delta-like 4 factor (Dll-4), an important factor that regulates angiogenesis, was present into EXOs released from ECs overexpressing Dll-4, and that these EXOs were taken up by the neighboring ECs. The transfer of these EXOs through ECs promoted the increase in angiogenesis by inhibiting Notch signaling, without requiring cell–cell contact [29]. Another factor delivered by EC EXOs, which resulted implicated in the modulation of angiogenesis and vessel formation, is hsa-miR-214 [30]. Van Balkom and colleagues showed the transfer of this miRNA between ECs and demonstrated that this caused the downregulation of ataxia telangiectasia mutated (ATM) in recipient cells. ATM is responsible for the prevention of cell-cycle progression; thus, its downregulation means a repression of cell senescence and an induction of the angiogenetic program [30].

In particular conditions such as peripartum cardiomyopathy, ECs, stimulated by 16-kDa N-terminal prolactin fragment (16K PRL), overexpress miRNA-146a that exerts anti-angiogenic and anti-proliferative effects on ECs. This miRNA is also secreted through EXOs release by ECs that are efficiently taken up by CMs, thus modulating their activity [31]. Halkein et al. demonstrated that EC EXOs, enriched in miR-146a, taken up by CMs, promoted the decrease of CM metabolic activity and downregulated Erbb4, Notch1, and Interleukin-1 receptor associated kinase 1 (Irak1) expression, proteins which are normally upregulated in the maternal heart, leading to the development of peripartum cardiomyopathy [31].

### 2.4. Cardiac-Derived Progenitor Cells and Cardiosphere-Derived Cells

Several studies demonstrated that the adult heart contains a group of heterologous cells, senescent in physiological conditions, capable of being activated by injuries and of differentiating into new myocytes or vascular cells; these cells are named cardiac-derived progenitor cells (CPCs) [32,33]. These cells spontaneously diffuse out from ex vivo cultures of heart tissue. When cultured in suspension, CPCs have the tendency to form spherical aggregates, denominated cardiospheres (CDCs). These aggregates differ from CPCs and present different properties [20].

It was shown that EVs can be released from CPCs and CDCs, and it was shown that EXOs were the predominant fraction of EVs [34,35]. In 2014, Barile et al. studied the effects of conditioned medium from CPCs, which contained EXOs, on the HL-1 cardiomyocytic cell line and on human umbilical vein endothelial cells (HUVEC), finding that it decreased HL-1 cell apoptosis and promoted tube formation in the HUVEC culture. MicroRNA transcriptional profiling of EV content underlined an enrichment in hsa-miR-210, hsa-miR-132, hsa-miR-146a-3p, and hsa-miR-181, compared with the profiling of fibroblasts EVs [36]. In particular, Barile and colleagues hypothesized that the presence of a high amount of hsa-miR-210 sustained the anti-apoptotic effect, as it is associated with the downregulation of its targets ephrin A3 and Protein-tyrosine-phosphatase 1 (PTP1), while the increased presence of hsa-miR-132, associated with the functional downregulation of its target RasGap-p120, was indicated as responsible for the angiogenetic effects [36]. The protection of CMs from apoptosis by CPC EXOs could be sustained also by the content of mmu-miR-21, which seemed to downregulate programmed cell death 4 (PDCD4) expression; indeed, this factor is involved in the miRNA-21/PDCD4 axis that importantly mediates CM apoptosis induced by oxidative stress [37].

A recent study suggested that the cardioprotective capacity of CPCEXOs could be due also to the presence of pregnancy-associated plasma protein-A (PAPP-A) on their surface [38]. The active form of this protein cleaved Insulin-like growth factor binding protein-4 (IGFBP-4) promoting the release of Insulin-like growth factor-1 (IGF-1), a key cardioprotective factor. Moreover, EXOs from CPCs favored the phosphorylation of Insulin-like growth factor receptor (IGFR) and intracellular Extracellular-signal regulated kinase 1 and 2 (Erk1/2) in CMs treated with staurosporine [38]. 

**Figure 5:**
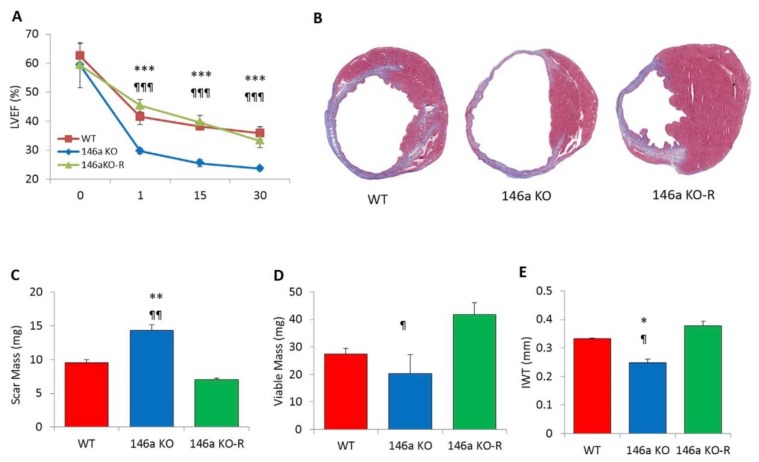
Involvement of microRNA (miR)-146a in the impairment of heart function after myocardial infarction (MI). (**A**) Measurement of left-ventricular ejection fraction indicates that 146a knockout (KO) animals have severely impaired cardiac function and structure following acute MI compared to wild-type (WT) or 146a KO-R mice. (**B**) Representative Masson’s trichrome-stained sections of hearts from three groups show the different tissue regeneration after MI. (**C**–**E**) Morphometric analysis reveals impairment of cardiosphere (CDC)-mediated benefits as evident in pooled data for scar mass, viable mass, and infarct wall thickness (IWT) in hearts of 146a KO mice. * *p* < 0.05, ¶ *p* < 0.05; ** *p* < 0.01, and ¶¶ *p* < 0.01, *** *p* < 0.001, and ¶¶¶ *p* < 0.001 using Student’s *t*-test (* KO versus WT; ¶ KO versus KO-R). Data are represented as means and standard errors of the mean (SEM). Modified from Reference [39].

Regarding CDCs, Ibrahim and colleagues also showed that EXOs derived from them had cardioprotective function. They resulted enriched in hsa-miR-146 compared to those of the fibroblasts [39]; this miRNA has known targets Irak1 and Traf6, two signaling mediators of the Toll-like receptor (TLR)–NF-κB axis [40,41]. In this study, the exposure of CMs to an miR-146a mimic promoted their protection against oxidative stress and, as a consequence, CM viability increased. Furthermore, the knockout of miR-146a in mice (146a KO) significantly impaired heart function after myocardial infarction (MI), also causing adverse tissue remodeling compared to wild-type (WT) mice or miR-146a KO mice injected with an miR-146a mimic (146a KO-R) [39] (Figure 5). The authors concluded that this evidence pointed to miRNA-146a potentially mediating the benefits of CDC EXOs. 

Most of the studies that investigated the role of CPC EXOs on different populations of infarcted heart cells did not take in consideration that, in physiological conditions, CPCs are also exposed to hypoxic conditions. In their work, Gray et al. paid attention to this and utilized EXOs from CPCs exposed to 3 h or 12 h of hypoxia in treated ECs and CFs. They pointed out that 12-h hypoxic CPC EXOs significantly enhanced tube formation in ECs and decreased pro-fibrotic factor expression in CFs, while 3-h hypoxic CPC EXOs or normoxic CPC EXOs did not have such pronounced effects [42]. Interestingly they saw that hypoxic EXOs were poor in miRNA-320, miRNA-222, and miRNA-185 content, correlated with anti-angiogenic, pro-apoptotic and anti-migration, and pro-fibrotic effects, respectively [42].

At the beginning of 2018, Nie et al. published a paper in which they performed proteomic and RNA sequencing analysis of the CPC secretome [43]. The results were in agreement with previous studies and confirmed the pro-survival, pro-angiogenic, and pro-mitogenic effects of CPC EXOs content. They found elevated levels of miRNA precursors and miRNAs that stimulate cell survival, proliferation, and angiogenesis, such as hsa-miR-3615, hsa-miR-6087, hsa-miR-1244, and hsa-miR-3687, and, in addition to these, small and long non-coding RNAs. Interestingly, proteomic analysis revealed that, within the EVs, 93% of proteins had their corresponding mRNA, suggesting that they were captured during the translation process. Consequently, proteins also resulted to be implicated in cell survival, proliferation, and angiogenesis [43].

## 3. Exosomes as Biomarkers in Cardiovascular Diseases

In addition to being internalized by neighboring cells, EXOs produced by cardiac cells are released into the body fluids. This allows exploiting EXOs as biomarkers that indicate a pathological state, considering that EXO content could vary in relation to it.

MicroRNAs are the most studied elements contained in EXOs for their role as new biomarkers in cardiovascular diseases. The majority of miRNAs isolated from plasma contained EXOs and bound to RNA-binding proteins, while only few miRNAs were free [44]; through analyzing the content of circulating EXOs, it was and it will possible to identify miRNAs that, changing in quantity, can be considered as biomarkers.

Focusing on cardiovascular diseases, different miRNAs were individualized for this purpose; for example, hsa-miR-1 and hsa-miR-133a, two cardiac-specific miRNAs, were demonstrated to be upregulated in serum from patients with acute coronary syndrome (ACS), and they were very likely stored in EXOs [45].

In acute pathologies, such as ACS and acute myocardial infarction (AMI), it is very important to rapidly individualize them. The classical biomarkers used to diagnose AMI are troponin and creatinine kinase MB, which sometimes coordinate with other biomarkers. Troponin levels peak at 12 h from the onset of cardiac damage, and their levels are proportional to the infarct size [46]. Recently, circulating miRNAs were discovered that were upregulated in the plasma of AMI patients, which reached their peak level earlier then troponin. An example was hsa-miR-208a; it was undetectable in healthy patients, but clearly appeared in 100% of AMI patients after 4 h from the onset of chest pain, very early compared to the appearance of detectable traces of troponin [47]. From the same family, hsa-miR-208b was also evaluated as an AMI biomarker. It was found that this miRNA was significantly increased in AMI patients within 12 h, making it a potentially good biomarker, but not one superior to troponin as they have a similar trend. The same consideration was made for hsa-miR-1, hsa-miR-133a, and hsa-miR-499 [48]. A research by Gidlöf et al. showed that the upregulation of plasma levels of hsa-miR-208b and hsa-miR-499-5p corresponded to an increase in the risk of death or heart failure, giving an indication of the prognosis [49]. Matsumoto and colleagues found that three particular p53-responsive miRNAs enriched in circulating EXOs, hsa-miR-192, hsa-miR-194 and hsa-miR-34a, were upregulated in the serum of AMI patients that experienced development of heart failure within one year, leading them to be considered as possible prognostic markers [50].

Few studies analyzed the proteomic profile of EXOs found in the bloodstream of MI patients. Cheow et al. identified six novel proteins that might be biomarkers of myocardial injury; these belonged to complement activation (C1Q1A and C5), lipid metabolism (APOD and APOC3), and platelet activation pathways (GP1BA and PPBP) [51].

Altogether, the studies reported above indicated that the heart releases characteristic EXOs following the onset of injury. The content of these EXOs might be useful for an early diagnosis and for hypothesizing, and thereby trying to prevent, a future prognosis. Some elements seem to be able to help formulate a diagnosis earlier than those actually used, while others, added to the classical analysis, could serve to pronounce a more precise diagnosis.

## 4. Exosomes as Therapeutic Agents in Cardiovascular Diseases

The possible use of EXOs as a substitute to whole-cell therapy received great interest. EXOs, in fact, possess several advantages compared to cells for therapeutic use; they are biocompatible, non-immunogenic, and non-tumorigenic; moreover, they are physiologically more stable than cells, can circulate all over the body, and are able to cross blood–brain barrier (BBB). Moreover, they are suitable to be loaded with therapeutic cargos, and they are more resistant to freezing and thawing procedures than cells, favoring long-term storage [17].

As reviewed before, several studies were conducted to understand EXO function in cardiovascular physiology and pathology. Through these researches, it emerged that EXOs generated from different cardiac cell types in different conditions could contain cargo that generates positive or negative effects on target cells. This gave some ideas on how to exploit EXOs for therapeutic approaches, which can be divided into two big categories: those that counteract adverse functions of harmful EXOs, and those that take advantage of and enhance the cardioprotective effects of beneficial EXOs.

### 4.1. Strategies to Attenuate Adverse Effects of Exosomes

As already mentioned, it was discovered that some factors contained in EXOs generated in pathological conditions have a detrimental role in cardiovascular diseases.

Studies on EXO biogenesis and trafficking gave suggestions on how to attenuate EXO adverse effects during diseases, starting from their formation and ending with their release and uptake from target cells.

To block EXO formation, it was found efficient to inhibit ceramide formation using inhibitors of neutral sphingomyelinases, such as GW4869, [6], or using amiloride, an anti-hypertensive drug that blocks Ca^2+^-dependent MVB formation [52]. Another way seemed to be the inhibition of the interaction between syndecan proteoglycans and its cytoplasmic adaptor syntetin, which interacts with programmed cell death-6-interacting protein (PDCD6IP or ALIX), an important protein involved in EXO biogenesis [53].

Regarding EXO release and uptake from target cells, the various mechanisms through which they occur are not yet detailed, and the fact that they differ through cell types makes them a difficult target to approach. Moreover, most studies were performed on tumor cells that represent a very particular condition, making them difficult to transfer to the cardiovascular field.

All these pathways offer many opportunities to look for right ways to inhibit harmful EXOs; however, at the same time, interfering with these processes, fundamental also for the regulation and the maintenance of physiological state, could be dangerous because of off-target effects.

### 4.2. Strategies to Exploit Exosomes in Cardiovascular Therapy

In the last few years, stem-cell therapies caught attention in many research fields, and were found to be successful in many cases. However, in the cardiovascular field, they did not appear so promising; they displayed poor engraftment and survival, as well as the occurrence of arrhythmias and immune rejection after transplantation [54].

Increasing evidence demonstrated that several cell types, above all stem cells, display their paracrine effects through EXO release. This shifted the focus from cell therapy to cell-free therapy approaches, and stem cells represent the most promising EXO source for cardiovascular therapy.

For investigating cardioprotective and cardiac repair effects of EXOs, scientists isolated them from stem cells of different origin: mesenchymal stem cells (MSCs), induced-pluripotent stem cells (iPSCs), and CPCs. Arslan et al. were among the first to investigate the role of MSC EXOs on myocardial ischemia/reperfusion injury, finding that EXO treatment activated pro-survival signaling by increasing ATP and Nicotinamide adenine dinucleotide (NADH) levels, and decreasing oxidative stress. Moreover, cardiac function was enhanced and infarct size was reduced [55]. Feng and colleagues showed that MSCs that underwent ischemic preconditions produced EXOs able to improve cardiac function after MI, by reducing infarct size and fibrosis [56]. Another work explained that EXOs derived from MSCs overexpressing GATA-4 resulted enriched in rno-miR-19a, which could be the promoter of increased CM survival in a hypoxic environment [57].

As already mentioned, EXOs from CPCs also resulted having regenerative potential after myocardial infarction. Several works demonstrated that they reduce CMs apoptosis, increase tube formation, and decrease the development of fibrosis. This leads to an improvement in cardiac function [36,39,42]. Interestingly, the pretreatment of CPCs with MSC EXOs seems to modify CPC EXO cargo so as to increase in rno-miR-147 and rno-miR-503-3p levels, and decrease in rno-miR-207, rno-miR-326-5p, and rno-miR-702-5p levels. These cells, once injected, were shown to promote an increase in vessel density at the infarct site and to ameliorate cardiac function [58]. When stimulated with embryonic system-cell (ES) EXOs, CPCs express more CM and EC genes. The injection of pre-stimulated CPCs resulted in increased cardiac function and reduced infarct size, while the direct injection of ES MVs/EXOs increased vessel density and improved cardiac function [59].

These studies suggest that changing donor cell conditions is a valid way of modifying EXO content, potentiating their cardiovascular protection ability. In addition, pretreatment of stem cells with EXOs derived from other cells can enhance their therapeutic effects.

A curious and unusual study was performed by Middleton and colleagues, who took a cue from newts, as they can regenerate lost organs and tissues, including the heart, and analyzed the compatibility of newt EVs with mammalian cells [60]. They extracted EVs form newt myogenic precursor cells (A1) and, after seeing that, in many ways (size, morphology, content, surface agents, and GW4869 sensitivity), they were similar to mammalian ones, they treated mammalian CMs with these vesicles. Gene expression analysis indicated that treated CMs were more resistant to oxidative stress and, thus, had enhanced cell survival, due to the activation of the Protein kinase B (AKT) pathway [60]. This evidence makes A1 EXOs and their content interesting candidates for therapeutic studies in cardiovascular fields.

**Figure 6:**
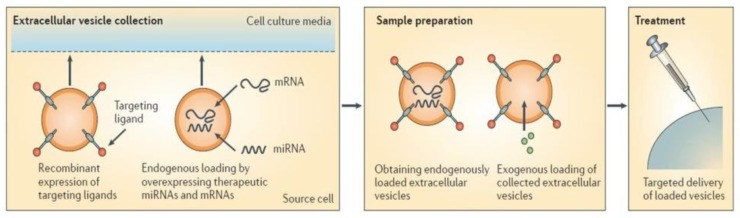
Engineering of extracellular vesicles (EVs). EVs can be engineered to load desired items, which can be carried out either endogenously or exogenously. Endogenous loading is achieved via transfecting parent cells strongly overexpressing miRNA or mRNA; this results in the production of EXOs that are already loaded with the elements of interest upon their collection. Exogenous loading allows the collection of drug-free EXOs which are then loaded with desired cargo molecules either via simple co-incubation with suitable cargo molecules or via active encapsulation with the help of certain procedures, such as electroporation. Modified from Reference [62].

In addition to the natural effect of stem-cell EXOs, methodologies for loading non-native cargo continue to be investigated to extend their therapeutic potential (Figure 6). Currently, three main strategies are available: endogenous encapsulation, and passive or active exogenous cargo encapsulation. Endogenous loading plans to modify parent cells via transfection or specific treatments, whereby derived EXOs will reflect the change in parent cells, and part of them will contain the loaded elements [61,62]. Passive loading methods include the simple incubation of EXOs with drugs that diffuse into them along the concentration gradient [63]. Instead, the possible methods for the active encapsulation of selected cargo involve sonication, extrusion, or electroporation [64,65,66]. These methods temporally perturb the EXO lipid bilayer, allowing drugs to enter them.

These various approaches have different loading yields, which obviously depend on the properties of the cargo. The application of these methodologies in cardiovascular diseases is still in its infancy, but the results obtained treating other pathologies such as cancer generate great expectations.

## 5. Exosome Delivery to Target Cells

Despite the many advantages of the possibility of using EXOs as therapy in cardiovascular disease, a determining factor to make this possible is finding the right way to deliver them so they are effective.

For cardiovascular therapy, the ideal modality to deliver EXOs is obviously intravenous injection; however, it was demonstrated that this way predominantly led to absorption within the liver [67]. It was then tried to execute intramyocardial (IM) and intracoronary (IC) injections; the comparison between these two ways highlighted that EXO IM injection was more effective than IC injection [68]. Gallet and colleagues demonstrated that CDC EXO IM injections delivered in infarcted pig heart showed greater myocardial retention and, as a consequence, a significant decrease in scar size and in microvascular obstruction compared to IC delivery [68].

Vandergriff et al. highlighted that IM delivery in clinical practice is not the most eligible method because of its correlated risks [69]. To try solving the problem, they resorted to the modification of the EXO surface, a heavily researched area which aims to improve targeting of EXOs to cells of interest. In particular, they tried tagging EXOs with cardiac-homing peptide (CHP), a peptide that targets infarcted heart, and to inject them into the tail vein of infarcted rats [69]. This functionalization improved cardiac EXO retention, leading to the induction of CM proliferation, an increase in angiogenesis, and a reduction in heart fibrosis of infarcted rats [69]. In another study, Kim and colleagues generated EXOs that expressed cardiac-targeting peptide (CTP) bound to Lysosomal-associated membrane protein 2b (Lamp2b), an exosomal membrane protein, on the surface [70]. In vitro and in vivo experiments showed that CTP EXOs were preferentially delivered into heart cells and tissue, with an increase of 15% [71]. A peptide sequence, CSTSMLKAC, named ischemic myocardium-targeting peptide (IMTP), which targets ischemic areas of the heart, was discovered by Kanki et al. [71], and was utilized by Wang and colleagues to engineer the MSC EXO surface such that its cargo was released into injured CMs [72]. Their hypothesis was confirmed by in vivo experiments that highlighted a significant increase in targeting ischemic myocardium by IMTP-modified EXOs, leading to a significant improvement in cardiac health [72].

High retention in the liver is one of the biggest obstacles related to drug delivery, and EXOs addressed to the heart are not exempt from this problem. However, engineered EXOs seem to significantly improve the amount that reaches cardiac target cells.

## 6. Conclusions

The intent of this review was to take stock of the current state of the art regarding EXOs in cardiovascular diseases. This topic is of the great interest because the study of EXOs is revealing a lot of new information about paracrine cell communication, which is proving to be fundamental for the maintenance of physiologic organ homeostasis. Early studies immediately created great prospects for EXO use as therapy in many diseases, including cardiovascular ones. It soon became clear that they are advantageous compared to cell therapy, especially in this field, where whole-cell therapy seemed not promising [54]. Apart from their native properties, which can be exploited in therapy, they were shown to be suitable for modification so as to target drugs for specific cells and for use as biomarkers [17,46,47,48,49,50,51].

Study of the cardiac environment showed an amazing communication network, which exploits EXOs, between the various cardiac cells. This communication through EXOs was implied in the maintenance of cardiac homeostasis, but most of all in the adaptive response to stress signals. It follows that EXOs are involved in many cardiovascular process, having the capability to improve or worsen cardiac health [10,11,12,13]. For these reasons, many studies on EXO functions in cardiovascular disease continue to be carried out with the hope of exploiting them for developing new therapies for many cardiovascular pathologies.

In conclusion, this review highlighted that there is still a lot of work to be done before there are any real opportunities to use EXOs to treat cardiovascular diseases; standardized high-yield and non-expensive protocols to isolate and characterize EXOs remain to be developed. Furthermore, their susceptibility to change makes it difficult to set up scalable and reproducible isolation processes. There are also no exhaustive studies about EXO kinetics, and many other points need to be deeply investigated. Instead, their use as biomarkers in the individualization of cardiac acute events might be a closer goal.
